# Watt’s Crystal Gold

**Published:** 1893-05

**Authors:** Frederick S. Hopkins


### WATT'S CRYSTAL GOLD.[1]

BY FREDERICK S. HOPKINS, D.M.D.

WATT'S crystal gold made its appearance many years ago, but it was very far from being the perfect article which we now have.

We often hear old practitioners say, "Crystal gold? oh, yes, I used to make it."

---

[1] Read before the Harvard Odontological Society, November 30, 1892.

He made it, used it for a time, and discarded it, because it failed to accomplish what it promised. This home-made article worked well, but soon disintegrated, pitted and flaked away, so that one trial was enough, and ever since crystal gold has been to him a synonyme for failure.

The simple mechanical ability to pack gold well does not in itself guarantee a perfect filling; and, although it is often said that it makes little difference what gold a good operator uses, yet we are continually finding gold fillings, put in by first-class operators, which have failed, not because the gold was not hard and well condensed, or through any faulty mechanical manipulation, the filling itself bearing every appearance of finished workmanship. Then why should it fail? Why should the tooth decay at the cervical margin any more with gold than with gutta-percha? Certainly not because of any peculiar antiseptic or preserving quality of the gutta-percha, but for the simple reason that, being plastic when put in, it makes an absolutely tight stopping.

Some one has made the statement that it was impossible to make an absolutely tight filling with cohesive gold. If he had said cohesive gold-foil, his statement would, perhaps, have been more nearly correct.

A filling does not necessarily need to be rocking or show discoloration around it to allow moisture to enter between it and the walls of the cavity. If you could examine the best of prepared cavities before the gold was placed in it, with a good magnifier, you would find not a clean, smooth surface, but a perfect labyrinth of flaws, scratches, and pits, which would show at once that nothing but a plastic material could fill them, and certainly not gold-foil. Unless every atom of exposed surface of that cavity comes into contact with the filling material, you will have numerous catch-basins in which decay may start, and often does.

Watt's gold, being made up of fine crystals, which have been left in almost the very condition in which they were deposited, and not having received the hammering or rolling which foil gets, retains its maximum degree of softness and adaptability with the greatest possible amount of cohesiveness, which are the qualities most sought for in cohesive gold. While, perhaps, it will not perfectly fill all the defective places in the prepared cavity, it will pack down into them and the retaining-grooves, where foil would bridge over, no matter how carefully condensed.

In this gold we find the fibre-like crystals running in all directions, and when packed hard it makes the toughest of fillings.

In some places, some of the so-called plastic golds might do as well, perhaps better, but in retaining-grooves and places where strength is demanded they do not, because they lack this fibrous quality, and fracture too easily.

Crystal gold is, as ordinarily used, a slow gold to work, but, if annealed in mica, it can be picked up on the plugger-point much more readily, and with very much less condensation, so that you dispense with the movement of carrying the gold to the flame and then to the cavity, and have your gold in much better condition when it is placed, so that, working it in this way, it is about as rapid as cohesive foil.

If you wish to carry more than one piece to the cavity, the slightest touch of the piece already on your plugger-point will carry it along. It should not be annealed in the naked flame, for the crystals are instantly melted and its peculiar softness destroyed, and any attempt at stuffing will most surely result in failure, for, although it may have the appearance of being well condensed, it is so only at the surface, underneath being soft and crumbling.

In small cavities it is especially useful on account of its great softness and adaptability, packing down with that dead, lead-like quality which makes it stay where it is put. In small, inaccessible approximal cavities, where there is scarcely room for the plugger-point, it can be tucked in with the point of the plugger where foil would be partially condensed before it reached the cavity. It is often convenient to start a gold filling in soft cement, and here the fibrous quality of the gold enables you to pack it in with great ease and without forcing the cement into your retaining-grooves, as you would be likely to do with the smooth-surfaced foil. You can use a fine, sharp point, and prick the gold several times into the soft cement, which will retain it finely, when, after the cement hardens, the gold may be packed down more solidly with a larger point, making a base which is firm and solid.

In combination fillings of gold and amalgam, in which the gold is packed upon the amalgam while soft, we have nothing which compares with this gold as a starter in the shape of foil. Some of the other forms of precipitated gold do well here, but no better.

Occasionally we have a case where the patient comes in with a portion of a large contoured gold corner of a central or lateral incisor broken, and the remaining portion is solid, well condensed, and otherwise in good order, and we wish to restore the contour.

The surface of the broken part may be freshened, made clean with chloroform, and annealed, when small pieces of Watt's crystal gold will cohere with a tenacity which, I think, is impossible to attain with any other form of gold.

This gold comes prepared in several different degrees of condensation, but the number one, or the lightest, works best, the other numbers being apt to get too much condensed in handling, and the pieces which are picked off too thick. It is not a gold to be used to the exclusion of all others, but in certain places its peculiar qualities make it much superior to foil.

